# Economic Evaluation Enhances Public Health Decision Making

**DOI:** 10.3389/fpubh.2015.00164

**Published:** 2015-06-24

**Authors:** Kristina M. Rabarison, Connie L. Bish, Mehran S. Massoudi, Wayne H. Giles

**Affiliations:** ^1^Division of Population Health, National Center for Chronic Disease and Health Promotion, Centers for Disease Control and Prevention, Atlanta, GA, USA

**Keywords:** economic evaluation, cost analysis, cost-effectiveness analysis, cost-benefit analysis, cost-utility analysis, public health economics, public health leadership

## Abstract

Contemporary public health professionals must address the health needs of a diverse population with constrained budgets and shrinking funds. Economic evaluation contributes to evidence-based decision making by helping the public health community identify, measure, and compare activities with the necessary impact, scalability, and sustainability to optimize population health. Asking “how do investments in public health strategies influence or offset the need for downstream spending on medical care and/or social services?” is important when making decisions about resource allocation and scaling of interventions.

In 2012, the United States (U.S.) total health care spending was $2.8 trillion (1). Most were used to treat diseases rather than prevent them, with only 2.7% dedicated to prevention (1). According to the National Association for County and City Health Officials and the Association of State and Territorial Health Officials, local and state health departments cut almost 60,000 public health jobs from 2008 to 2012 (2, 3). Contemporary public health professionals must address the health needs of a diverse population with constrained budgets and shrinking funds. It is critical for public health professionals to use a comprehensive approach to decision-making. This article’s aim is to provide a framework for use of economic evaluation by public health decision makers at the local, state, tribal, and national levels. We describe types of economic evaluation and provide examples of economic evaluation used by two public health research networks, the Centers for Disease Control and Prevention’s (CDC’s) Prevention Research Centers (PRCs) Program and Robert Wood Johnson Foundation (RWJF) funded Public Health Practice-Based Research Network (PH-PBRN).

## Evidence-Based Public Health and Decision Making

Public health professionals want to improve outcomes and minimize costs; evidence-based public health (EBPH) is integral in their decision-making process. EBPH is defined as “the development, implementation, and evaluation of effective programs and policies in public health through application of principles of scientific reasoning.” (4) EBPH uses the best available evidence, taking into consideration the population demographic characteristics, projected or tested program and intervention impacts, and estimated costs (5, 6). Understanding the economic evidence of public health intervention is an integral part of EBPH. Economic evidence can provide insight into the value of public health investments to the overall health system. Evidence suggests that increased investment in prevention activities and improvements in public health practice and decision making produce measurable and sustainable health gains (7). A study of local public health agencies in California from 2001 to 2008 found that a $10 per capita increase in public health investment could save 9.1 lives per 100,000 (8). This translates to 27,000 deaths per year averted with an economic value of $212 billion or more than $100 of benefit for $1 invested (8).

Public health professionals have become skilled at considering the epidemiologic evidence of health issues. Epidemiology is the cornerstone of public health, and informs policy decisions and evidence-based practice by identifying risk factors for disease and targets for preventive healthcare and interventions. But public health professionals also have to consider environmental constraints, such as funding and capacity, when choosing where to focus efforts. Economic evaluation provides evidence of the feasibility of intervention scalability and sustainability. Determination of the costs and benefits of public health interventions provides data for public health professionals and decision makers to use when choosing which interventions are effective, efficient, equitable, scalable, and sustainable (7, 9). Asking “how do investments in public health strategies influence or offset the need for downstream spending on medical care and/or social services?” (10) adds to informed decision making. Yet, economic evaluation remains a competency gap in public health decision making (11). To address this competency gap and prepare contemporary public health professionals, training for a public health profession might

- Offer elective courses on economic evaluation and public health economics in schools of public health
- Establish more post-doctoral trainings on economic evaluation and public health economics, such as the CDC Prevention Effectiveness Fellowship
- Include economic evaluation in Master and Doctor of Public Health (M.P.H. and Dr.P.H.) requirements and
- Provide continuing education for public health professionals at the local, state, tribal, and national levels through public health leadership institutes and training centers.

## Economic Evaluation

What is economic evaluation? By definition, economics is the study of decisions, through the examination of program incentives and consequences, and the measure of service production, delivery, and consumption (12). Economic evaluation is defined as “the systematic appraisal of costs and benefits of projects, normally undertaken to determine the relative economic efficiency of programs.” (13) Simply put, economic evaluation is the understanding and use of economic evidence in decision making.

Economic evaluation contributes to evidence-based decision making in public health by helping leaders and the community identify, measure, and compare activities with the necessary impact, scalability, and sustainability to optimize population health (13). In the words of Dr. Thomas Frieden, the Director of the U.S. Centers for Disease Control and Prevention, “to establish an effective intervention package, it is critical to understand the full range of available evidence-based strategies, the size and characteristics of the population to be reached, the projected impact of each intervention, and the estimated cost” (5).

## Types of Economic Evaluation and Decision Levels

There are two levels of economic evaluation: partial and full (Table 1). Partial economic evaluation measures program or disease costs, but does not involve a comparison with alternative options and does not relate costs to outcomes. Partial economic evaluations include cost-of-illness analysis and program cost analysis. In public health, full economic evaluation compares two or more public health interventions through the examination of costs of inputs and outcomes (14, 15). Full economic evaluations include cost-benefit, cost-effectiveness, and cost-utility analyses (14, 15).

**Table 1:** **Types of economic evaluation and decision levels**.

Type	Description	Measures	Decision level
**PARTIAL ECONOMIC EVALUATION**			
Cost of illness analysis	Disease economic burden	Net cost ($)	Public health decision-makers at the local, state, and national levels
Program cost analysis	Net program cost	Net cost ($)	Public health decision-makers at the local, state, and national levels
			First step to CEA, CUA, and CBA
**FULL ECONOMIC EVALUATION**			
Cost-benefit analysis (CBA)	Compares different programs with different outcomes (e.g., health vs. other area)	Benefit-cost ratio ($benefit: $cost)	National level and broader perspective, such as the President and Congress (e.g.,: Congress needs to decide between investments in health or investments for another program)
Cost-effectiveness analysis (CEA)	Compares interventions with the same outcomes (ex: between two cervical cancer interventions)	Cost-effectiveness ratio ($per case averted)	Program level (ex: a cancer program director decides to fund one of two possible cervical cancer prevention interventions)
Cost utility analysis (CUA)	Compares interventions with different health outcomes (ex: cervical cancer vs. Alzheimer’s disease)	Cost-utility ratio ($per QALY saved)	Agency level (ex: the CDC or a local health agency director decides between funding cervical cancer or Alzheimer’s disease interventions)

### Partial economic evaluation

- *Cost-of-illness analysis* estimates the economic burden or total costs attributable to a particular disease (14, 15). For example, the overall U.S. annual direct medical cost of preventing and treating HPV-associated disease was estimated at $8.0 billion (2010 USD); of this, total about $7.0 billion was spent on routine cervical cancer screening and treatment (16).
- *Program cost analysis* is a systematic collection and break down of the cost of a program with descriptions of who or what entity incurs which costs (14, 15). For example, the cost of a metformin intervention relative to a placebo intervention was $2,412 per participant, from a societal perspective over 3 years (17).

### Full economic evaluation

- *Cost benefit-analysis (CBA)* is considered the gold-standard of economic evaluation because all costs and benefits (and/or consequences – including health outcomes) are converted to a common metric such as dollars (14, 15). CBAs are used to decide among programs with different outcomes (14, 15). For example, the President, Congress, or a governor all might use CBA to decide between investments in health vs. another area. Benefit-cost ratio (BCR) is the summary measure of CBA. A 1.50:1 BCR means for every $1 of cost, society gains $1.50 of benefits (14, 15).
- *Cost-effectiveness analysis (CEA)* compares the costs with natural health outcome units, such as life-years saved and number of cases averted (14, 15). For example, a cancer prevention program director at a local health agency may need to decide between a number of interventions addressing the same health outcome. CEAs are appropriate to inform the decision because they maintain health outcomes in their natural units rather than monetize the outcome. Cost-effectiveness ratio (CER) is the summary measure of CEA results, and it is expressed in costs per natural health units such as dollars per life-year saved (14, 15). For example, the incremental CER of “Outcome Monitoring plus Recovery Management Checkups” of adults with chronic substance abuse in Chicago is $23.38 per abstinent day and $59.51 per reduced substance-related problem (e.g., liver disease) (18).
- *Cost-utility analysis (CUA)* is a special form of CEA where the costs and benefits (and/or consequences) are expressed as cost per a standardized morbidity and mortality measure, such as quality-adjusted life-year (QALY) (14, 15). QALY is a single measure of quality of life and survival (15). The summary measure of a CUA is expressed in cost per QALY (14, 15). A CUA is appropriate when making a decision at the agency level, such as the CDC or local health agency, where the director decides between public health interventions with different health outcomes. For example, the cost-utility ratios (i.e., cost per QALY) of population-wide strategies promoting physical activity in adults range from $14,000 to $69,000 per QALY gained (19).

## Application of Economic Evaluation in Public Health

Public health economic evidence can be found in The Cost-Effectiveness Analysis Registry from Tufts Medical School, (20) the National Health Services Economic Evaluation Database from the Cochrane Review, (21) and the Community Guide (22). In addition, the CDC’s State, Tribal, Local, and Territorial Public Health Professionals Gateway includes a collection of public health economics tools and methods (23). The role of economic evaluation in public health is gaining attention. However, it remains an under-used component of evidence-based public health decision making. In 2012, the Institute of Medicine Committee on Public Health Strategies to Improve Health Board on Population Health and Public Health Practice had some specific recommendations that are a call to action for the use of economic evidence in public health (24). These recommendations were:

- “Develop a model chart of accounts for use by public health agencies at all levels to enable better tracking of funding related to program outputs and outcomes across agencies”
- “Develop a robust research infrastructure for establishing the effectiveness and value of public health and prevention strategies”
- “Develop data systems and measures to capture research quality information on key elements of public health delivery including program implementation costs”
- “Develop and validate methods for comparing the benefits and costs of alternative strategies to improve population health.”

Since these recommendations were given, different efforts are underway to improve the use of economic evaluation in public health practice and decision making. Two examples of these efforts are the work done by two public health research networks: the CDC PRC program and the RWJF PH-PBRN.

### CDC prevention research centers program

The PRC program funds through cooperative agreements a national network of 26 academic research centers that are located at either a school of public health or a medical school that has a preventive medicine residency program. The PRCs are committed to conducting prevention research and are leaders in translating research results into policy and public health practice. The evaluation of the 2014–2019 PRC program added data collection to complete cost analysis of the core prevention research projects. As the Director of the CDC PRC program, Dr. Mehran Massoudi believes the cost analysis is important because “as scrutiny over federal spending increases with a greater sense of accountability, the PRC program has the unique opportunity to collect cost data associated with various facets of the PRCs’ research projects and in turn allow state and local health departments the ability to consider which components of the research projects can be adapted and or implemented for their use.” Dr. Wayne Giles, Director of the CDC’s Division of Population Health in the National Center for Chronic Disease Prevention and Health Promotion (NCCDPHP), added “As we work to improve the health and wellbeing of populations, it is increasingly important that our interventions demonstrate their ability to improve the health and delivery of care to populations while simultaneously addressing costs. Therefore, applied research documenting cost savings and return on investments are of high importance.”

The cost analysis will:

- Measure the actual expenditures related to the PRCs’ core research project
- Develop capacity of each institution and the PRC program to use economic evidence to assess the PRC’s research effectiveness, efficiency, equitability, scalability, and sustainability
- Provide baseline data for further economic evaluation studies, such as cost-effectiveness analysis and cost-benefit analysis.

A systematic collection of costs of a PRC’s core research project was done for the Rural Cancer Prevention Center (RCPC) at the University of Kentucky. The KY RCPC found that viewing the 1-2-3 Pap video increased the completion rate of human papillomavirus vaccination in 18- to 26-year-old women in Appalachian Kentucky. A hypothetical adaptation scenario showed that the intervention cost per completed three vaccination series would decrease from the efficacy study cost of $890 per completed series to an estimated implementation cost of $389 per completed series. Implementation cost estimates of the PRCs’ core research projects can provide additional evidence to public health practitioners and decision makers when assessing whether to implement and scale-up these projects in their communities.

Dr. Jeff Harris, the Principal Investigator for the University of Washington PRC, believes that “Economic evaluations are quite important for scaling up applied prevention research. In the short-term, public health managers need to know what an intervention will cost. In the long term, they need to know what an intervention will return.”

### RWJF public health-practice based research network

The PH-PBRN is a nationwide research network composed of local and state governmental public health agencies, community partners, and collaborating academic research institutions. Through the RWJF’s Delivery and Cost Studies (DACS) Program, 11 PH-PBRNs are conducting studies to estimate the cost of delivering public health services by examining how characteristics of public health delivery systems (e.g., activity scope, contributing organizations’ roles) impact the cost, quality, and equity of public health services delivery. For example, the Colorado PH-PBRN estimated the degree to which local public health structural differences impacted and changed the costs of delivering routine communicable disease surveillance by using a micro-costing economic evaluation method. Results showed that having a dedicated in-house communicable disease employee reduced spending by $138 per day or $50,370 per year.

## Conclusion

In todays’ economic climate of low resources and funding for public health programs, public health practitioners will benefit from the use of economic evaluations to enhance public health evidence-based decision making. Economic evaluation could provide data to help public health practitioners and decision makers to identify, measure, and compare a project’s resource allocation with the project’s impact, scalability, and sustainability to optimize population health.

As with epidemiology, economic evaluations are becoming another cornerstone in the foundation of public health decision making. When asked “why are economic evaluations important to public health,” Dr. Sam Posner, Associate Director of Science for CDC’s NCCDPHP, answered “Evaluation of public health interventions most commonly focuses on the impact on health outcomes and health status. Evaluating health impact is critical; however, it is equally important to conduct economic evaluations of interventions. For public health interventions to result in sustainable change, they need to both be effective in addressing the health burden and be economically defensible.”

The health of our population will benefit from assuring that future generations of public health professionals are educated and trained in economic evaluation. Now, and in the future, it is essential for public health professionals to understand and use economic evaluations as part of a comprehensive public health decision-making process.

## Conflict of Interest Statement

The authors do not have any conflict of interests to declare. This perspective article was written in the absence of any commercial relationships that could be construed as a potential conflict of interest. *Disclaimer*: the findings and conclusions in this paper are those of the authors and do not necessarily represent the official position of the Centers for Disease Control and Prevention.
